# Fra-1 affects chemotherapy sensitivity by inhibiting ferroptosis in gastric cancer cells

**DOI:** 10.20517/cdr.2024.101

**Published:** 2024-11-16

**Authors:** Feng Zeng, Jiaying Cao, Yan Chen, Jingqiong Tang, Qian He, Shan Liao, Lin Liang, Wentao Li, Siyi Liu, Gengqiu Luo, Yanhong Zhou

**Affiliations:** ^1^Cancer Research Institute, Basic School of Medicine, Central South University, Changsha 410011, Hunan, China.; ^2^Department of Geriatrics, The Second Xiangya Hospital of Central South University, Changsha 410011, Hunan, China.; ^3^Department of Radiation Oncology, Hunan Cancer Hospital & the Affiliated Cancer Hospital of Xiangya School of Medicine, Central South University, Changsha 410013, Hunan, China.; ^4^Department of Pathology, The Third Xiangya Hospital of Central South University, Changsha 410013, Hunan, China.; ^5^Department of Pathology, Xiangya Hospital, Basic School of Medicine, Central South University, Changsha 410008, Hunan, China.

**Keywords:** Gastric cancer, Fra-1, G6PD, pentose phosphate pathway, ferroptosis, chemotherapy resistance

## Abstract

**Aim:** Gastric cancer (GC) is one of the common malignant tumors, and most patients with advanced GC often develop chemotherapy resistance, resulting in poor chemotherapy efficacy. Therefore, it is crucial to clarify the specific mechanisms of their chemotherapy resistance.

**Methods:** In this study, we analyzed the correlation between fos-related antigen-1 (Fra-1) and chemotherapy resistance in GC using bioinformatics, cell counting kit-8 (CCK8), and 5-ethynyl-2’-deoxyuridine (EDU) combined with flow cytometry; furthermore, we used energy metabolomics sequencing, combined with ChIP-qPCR technology, to elucidate the specific role of Fra-1 in chemotherapy resistance of GC cells and its related mechanisms.

**Results:** We found that high Fra-1 expression was closely related to chemotherapeutic drugs in GC cells, as demonstrated by bioinformatics analysis combined with EDU and CCK8 experiments. Energy metabolomics combined with *in vitro* cellular experimental analysis revealed that the pentose phosphate pathway (PPP) was activated in GC cells with high Fra-1 expression, along with an increase in the synthesis of metabolites such as nicotinamide adenine dinucleotide phosphate (NADPH) and glutathione (GSH), a decrease in the level of reactive oxygen species (ROS), and the inhibition of their ferroptosis. In addition, ChIP-qPCR experiments confirmed that Fra-1 binds to the promoter of glucose-6-phosphate dehydrogenase (G6PD), a key rate-limiting enzyme of the PPP, and transcriptionally regulates its expression, which in turn activates the PPP and promotes chemotherapy resistance in GC cells.

**Conclusion:** Our research findings suggest that Fra-1 activates the PPP by upregulating G6PD transcriptional activity and inhibiting its ubiquitination level, inhibiting ferroptosis in GC cells and inducing chemoresistance. This provides an experimental basis for screening potential molecular targets for chemotherapy resistance in GC patients.

## INTRODUCTION

Gastric cancer (GC) ranks among the most prevalent malignant tumors globally, characterized by a low rate of early diagnosis and a dismal prognosis^[1]^. Chemotherapy stands as a primary treatment modality for the majority of patients with advanced GC. However, owing to the development of drug resistance, chemotherapy often yields poor efficacy, leading to a low 5-year survival rate among GC patients^[2]^. Chemotherapy resistance stems from multifaceted factors, resulting in a lack of specificity in target recognition^[3]^. Among these factors, ferroptosis emerges as a pivotal player in chemotherapy resistance. Ferroptosis represents a programmed cell death triggered by the accumulation of lipid peroxides and subsequent membrane damage. Activation of ferroptosis-related markers in tumor cells induces ferroptosis, mitigating tumor chemotherapy resistance, thereby enhancing chemotherapy efficacy^[4-6]^. For instance, studies have identified that tumors exhibiting high *TYRO3* gene expression manifest resistance to PD-1/PD-L1 blockade by impeding ferroptosis, resulting in resistance in syngeneic mouse models and patients receiving anti-PD-1/PD-L1 therapy^[7,8]^. These findings underscore the close association between ferroptosis and tumor chemotherapy resistance. Notably, there exist pertinent reports on ferroptosis and chemotherapy resistance in GC. For instance, cancer-associated fibroblasts impede ferroptosis in GC and foster chemotherapy resistance through the secretion of miR-522^[9,10]^. In summary, ferroptosis exerts a pivotal role in acquired drug resistance in GC. Thus, elucidating the role and mechanism of ferroptosis in GC chemotherapy resistance holds profound significance for enhancing the efficacy and prognosis of GC patients.

Fos-related antigen-1 (Fra-1), a member of the activator protein-1 (AP-1) transcription factor family, exhibits heightened expression in various tumors, correlating closely with cellular processes^[11-14]^. Our previous investigations have unveiled a robust expression of Fra-1, impacting the cell cycle distribution and apoptosis of GC cells, thereby contributing to the initiation and advancement of GC^[15]^. Furthermore, through immunoprecipitation coupled with liquid chromatography-tandem mass spectrometry, our group identified a novel interacting protein, YWHAH, associated with Fra-1. Subsequent mechanistic elucidation unveiled that YWHAH positively modulates Fra-1 expression, consequently activating the HMGA1/PI3K/AKT signaling cascade and fostering GC proliferation^[16]^. In essence, Fra-1 assumes a pivotal role in the malignant progression of GC. Nonetheless, to date, there exists a dearth of literature elucidating the involvement and underlying mechanisms of Fra-1 in GC chemotherapy resistance.

In this study, we have elucidated the underlying mechanism by which Fra-1 promotes chemotherapy resistance in GC. Our findings reveal that Fra-1 directly interacts with the promoter region of glucose-6-phosphate dehydrogenase (G6PD), the first rate-limiting enzyme in the pentose phosphate pathway (PPP), thereby transcriptionally upregulating its expression. Furthermore, Fra-1 enhances the stability of G6PD protein levels by inhibiting the ubiquitin-proteasome pathway. Consequently, this activation of the PPP leads to increased synthesis of the PPP metabolite nicotinamide adenine dinucleotide phosphate (NADPH) and a subsequent reduction in intracellular reactive oxygen species (ROS) levels. Ultimately, this cascade of events culminates in the inhibition of ferroptosis in GC cells, thereby conferring chemotherapy resistance in GC cells.

## METHODS

### Cell culture

The GC cell lines AGS and HGC27 were obtained from the Institute of Oncology, School of Basic Medical Sciences, Central South University, China. Both AGS and HGC27 cells were cultured in RPMI-1640 medium (Procell, PM150110, Wuhan, China) supplemented with 10% FBS (Procell, 164210-50, Wuhan, China). Chemosensitivity was evaluated in AGS and HGC27 cells treated with 10 μM cisplatin (CDDP) (MCE, 15663-27-1, New Jersey, USA), while ferroptosis was induced in AGS and HGC27 cells using 8 μM erastin (Selleck, S7242, Houston, USA). All cells were maintained at 37 °C with 5% CO_2_ and confirmed to be mycoplasma-negative.

### Vector

The following vectors were used: PLVX-mCMV-ZsGreen-PGK-Puro-Fra-1, a lentiviral system expression vector; Fra1-pCDNA3.1-3xFlag-C, a eukaryotic expression vector; G6PD-pCDNA3.1-3xFlag-C, a eukaryotic expression vector; TRIM21-pCDNA3.1-3xFlag-C, a eukaryotic expression vector; PSPAX2, a lentivirus packaging vector; PMD2.G, a lentivirus packaging vector; G6PD wild-type and mutant luciferase reporter gene plasmids: pGL3-Basic-G6PD-promoter-WT, pGL3-Basic-G6PD-promoter-MT; and PRL-TK, a eukaryotic expression vector. All constructs were selected for ampicillin resistance and screened with puromycin. They were purchased from biotech companies and confirmed through DNA sequencing. Antibodies and reagents used in the study can be found in Tables 1 and 2.

**Table 1:** Antibody information

**Name**	**Catalog No.**	**Ordering company**
GAPDH	YM3029	ImunnoWay
IgG	sc-34665	Santa Cruz Biotechnology
Goat anti rat IgG	RS0002	ImunnoWay
Goat anti mouse IgG	RS0001	ImunnoWay
HA-tag	YM3003	ImunnoWay
Flag	YM3808	ImunnoWay
Fra-1	YT1772	ImunnoWay
SLC7A11	YT8130	ImunnoWay
GPX4	YN3047	ImunnoWay
G6PD	YM0291	ImunnoWay
6PGD	YT3691	ImunnoWay
TKT	YT5938	ImunnoWay
TALDO	YT6489	ImunnoWay

G6PD: Glucose-6-phosphate dehydrogenase; 6PGD: 6-phosphogluconate dehydrogenase; TKT: transketolase; TALDO: transaldolase.

**Table 2:** Reagent information

**Name**	**Catalog No.**	**Ordering company**
Cycloheximide	HY-12320	MCE
MG132	S2619	Selleck
6AN	S9783	Selleck
Puromycin	IP1280	Solarbio
CDDP	15663-27-1	MCE
Erastin	S7242	Selleck

CDDP: Cisplatin.

### Cell viability assay (CCK8)

Cell viability was assessed using the CCK-8 reagent (Yeasen, 40203ES76, Shanghai, China) following the manufacturer’s instructions. Cells were seeded into 96-well plates and treated as indicated. After treatment, the culture medium was replaced with 100 μL of fresh medium per well, followed by the addition of 10 μL of CCK-8 solution. After 2 h of incubation at 37 °C with 5% CO_2_, the absorbance was measured at 450 nm using a microplate reader.

### Plasmid and siRNA transfection

For plasmid transfection, 2 μg of plasmid or shRNA was transfected into GC cells using polyplus transfection reagent (Sartorius Stedim Biotech, Aubagne, France) following the manufacturer’s instructions in six-well plates. Cells were harvested 48 h post-transfection for further analysis. For transient transfection of siRNA, 5 μL of siRNA was transfected into GC cells using polyplus transfection reagent according to the manufacturer’s instructions. A six-well plate was used as an example. The target sequence for siNC was 5’-ACGUGACACGUUCGGGAGAATT-3’, and for siFra-1 was 5’-GGAAGGAACTGACCGACTT-3’. All siRNAs were obtained from Guangzhou Ribobio Co., Ltd.

### RT-qPCR

Total RNA was extracted from cells using TRIzol (NCM Biotech, Beijing, China) and reverse transcribed into complementary DNA (cDNA) using the RNA First Strand cDNA Synthesis Kit (Yugong Biotech, Jiangsu, China). RT-qPCR assays were performed using the RT-qPCR Kit (SYBR Green method) (Yugong Life, Jiangsu, China) according to the manufacturer’s instructions. Relative gene expression levels were calculated using the 2^-ΔΔCt^ method with GAPDH as the internal reference^[17]^. Primer sequences are provided in Table 3.

**Table 3:** Primer sequence

**Gene name**	**Upstream and downstream primer sequences**
*Fra-1*	Forward 5‘-CAGTGGATGGTACAGCCTCATTTC-3’ Reverse 5‘-GCAGTCTCCTGTTCACAAGGC-3’
*G6PD*	Forward 5‘-ACACCAGGTATTTTGATGAGGAG-3’ Reverse 5‘-TCAGGCCGTGCCGCTGGCCGAGTAG-3
*6PGD*	Forward 5‘-AGCTGGTTTGGATCTTCGGA-3’ Reverse 5‘-CAGGTCATCCCCAGAGTTGT-3’
*TKT*	Forward 5‘-AGTTCATGTCACGCTGGGTA-3’ Reverse 5‘-CAGCTTCAGGTCTCCTTGGA-3’
*TALDO*	Forward 5‘-GCAACCCTTCTTTGACAACATTTTT-3’ Reverse 5‘-ATTTCTTCTCTCAGACGCTCTCC-3’
*SLC7A11*	Forward 5‘-GGCAGTGACCTTTTCTGAGC-3’ Reverse 5‘-TGTCAAAGGGTGCAAAACAA-3’
*GPX4*	Forward 5‘-GTAACCAGTTCGGGAAGCAG-3’ Reverse 5‘-TGTCGATGAGGAACTGTGGA-3’
*FTH1*	Forward 5‘-GGAGAGGGAACATGCTGAGA-3’ Reverse 5‘-TGTCGATGAGGAACTGTGGA-3’
*G6PD1*	Forward 5‘-CTCACTTCTGGTTCTGACCCC-3’ Reverse 5‘-TGACAATATGCGTGGAGCGG-3’
*G6PD2*	Forward 5‘-CCATGACCATGTTTGGTGTC-3’ Reverse 5‘-TGGACAGTAAGAGCGGAAGG-3’
*G6PD3*	Forward 5‘-TGACTTCCAGCAACACTGCC-3 Reverse 5‘-GGCCTCTGACATCAGTCACAA-3’
*G6PD4*	Forward 5‘-AATGGGGGTCATTTTTGTCA-3’ Reverse 5‘-TGCGAAGAAACTGGGAGAGA-3’
*G6PD5*	Forward 5‘-TGGTCACCTCACTCCACTCA-3’ Reverse 5‘-TCTCCTGGGAGTTCCTGATG-3’
*G6PD6*	Forward 5‘-CACCTGTGGTCCCAGCTACT-3’ Reverse 5‘-GACAGTTGCCTGATGGGTTC-3’
*GAPDH*	Forward 5‘-TCAAGAAGGTGGTGAAGCAGG-3’ Reverse 5‘-TCAAAGGTGGAGGAGTGGGT-3’

Fra-1: Fos-related antigen-1; G6PD: glucose-6-phosphate dehydrogenase; 6PGD: 6-phosphogluconate dehydrogenase; TKT: transketolase; TALDO: transaldolase.

### Western blot

Total proteins were extracted using RIPA lysis buffer (Beyotime) containing protease inhibitors, and protein concentrations were determined using a BCA protein assay kit (Thermo Fisher Scientific). Proteins were separated by SDS-PAGE and transferred to PVDF membranes (Millipore, Bedford, MA, USA). Membranes were blocked with 5% skimmed milk and then incubated with primary antibodies overnight at 4 °C. After incubation with secondary antibodies, protein bands were visualized using an ECL detection reagent (NCM).

### ChIP-qPCR

ChIP assays were performed using the ChIP assay kit (Beyotime, P2078, Wuhan, China) following the manufacturer’s protocol. Chromatin immunoprecipitation was performed, and gene enrichment was detected using RT-qPCR.

### Dual luciferase reporter assay

Luciferase activity was measured using a dual luciferase reporter assay (Uelandy, F6075S, Suzhou, China) according to the manufacturer’s instructions. Fluorescence values were determined using a microplate reader.

### Co-IP

Co-IP assays were conducted by lysing cells and immunoprecipitating the target proteins using magnetic beads conjugated with specific antibodies. Protein interactions were detected by SDS-PAGE electrophoresis.

### Immunohistochemistry

Immunohistochemistry was performed on FFPE sections from mouse subcutaneous tumor tissues using specific primary antibodies. The staining intensity was scored, and images were analyzed.

### Nude mouse experiment

Twenty 4-week-old female B/C nude mice were ordered from Hunan Slake Jingda Laboratory Animals, Ltd. and maintained in pathogen-free conditions. All animal experiments were conducted in accordance with the guidance of the Animal Ethics Committee of Central South University and approved by the Experimental Animal Welfare Ethics Committee of Central South University (approval no. CSU-2023-0337; Changsha, China). First, 20 mice were randomly divided into 4 groups. Subcutaneous tumorigenic experiments were then performed (injection of 5 × 10^6^ HGC27 cells successfully overexpressed Fra-1). Tumor size was monitored every 3 days using the formula: (longest and shortest diameter^2^) × 0.5^[18]^. Mice were euthanized after the last measurement, and the tumors were collected for further studies.

### Statistical analysis

All experiments were repeated at least three times, and data were expressed as mean ± standard deviation. Statistical analyses were performed using GraphPad Prism 9, with Student’s *t*-test or ANOVA used for comparisons between groups. *P* < 0.05 was considered significant (ns, not significant, ^*^P < 0.05, ^**^P < 0.01,^***^P < 0.001, ^****^P < 0.0001).

## RESULTS

### Fra-1 is highly expressed in GC tissue and is closely related to chemotherapy resistance and poor prognosis in GC cells

**Figure 1:**
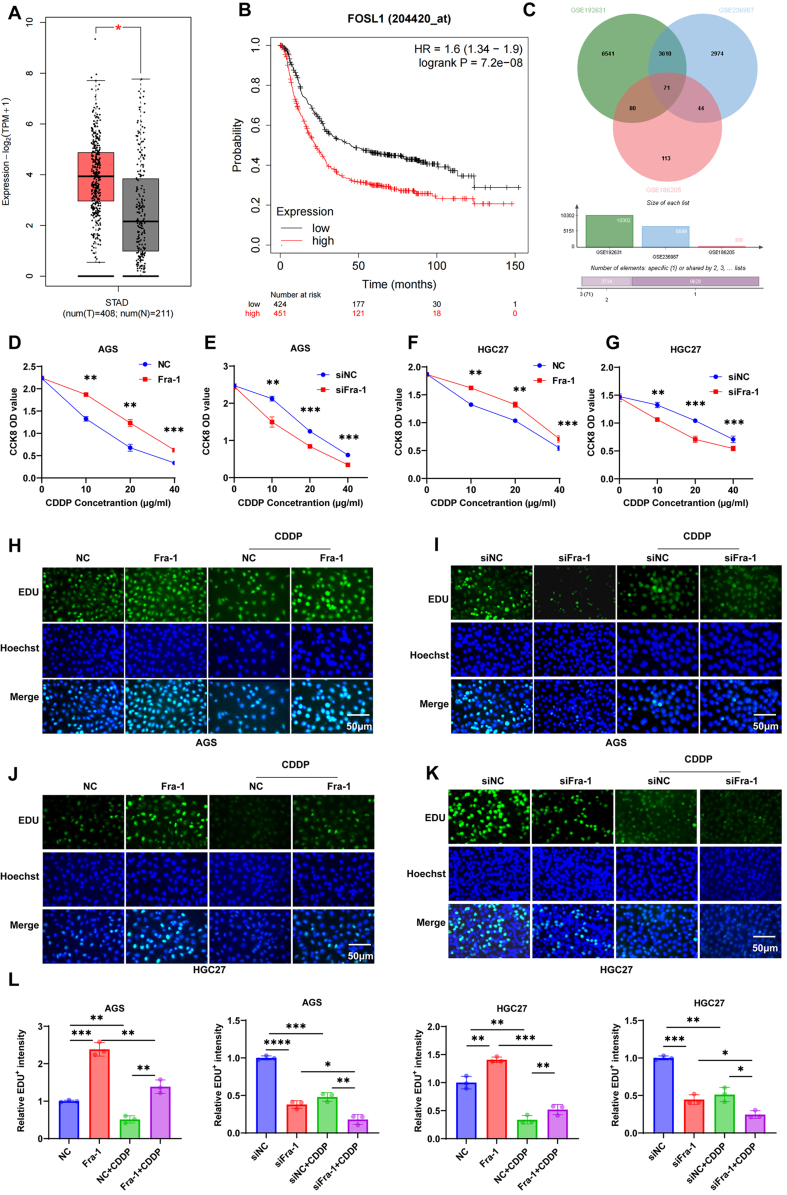
Fra-1 is closely associated with chemoresistance in GC. (A) Fra-1 expression in the STAD dataset analyzed using box plots from the GEPIA2 database. T, tumor samples (*n* = 408 cases); N, normal samples (*n* = 211 cases); (B) Kaplan-Meier Plotter survival curves showing the distribution of OS in GC patients with high/low expression of Fra-1 in the STAD cohort; (C) Wayne plots demonstrating differentially expressed genes common to GC drug resistance datasets GSE192631, GSE236987, and GSE186205; (D-G) Cell proliferation ability of GC cells after overexpression/silencing of Fra-1 and treatment with CDDP detected using the CCK8 Cell Proliferation Detection Kit; (H-K) Cell proliferation ability of GC cells after overexpression/silencing of Fra-1 and treatment with CDDP (10 μM) detected using the EDU cell proliferation detection kit. Scale bar = 50 μm; (L) Statistical graph of semi-quantitative results of EDU fluorescence values in GC cells. All experiments were performed with three technical replicates. ^*^P < 0.05; ^**^P < 0.01; ^***^P < 0.001. Fra-1: Fos-related antigen-1; GC: gastric cancer; OS: overall survival; CDDP: cisplatin; CCK8: cell counting kit-8; EDU: 5-ethynyl-2’-deoxyuridine.

To delve deeper into the mechanistic insights underlying Fra-1’s impact on GC progression, we initiated an integrated analysis utilizing the GEPIA2 database, where we observed elevated expression levels of Fra-1 in GC tissues [Figure 1A]. Subsequent analysis through the DRESIS database further corroborated this observation, indicating a positive correlation between Fra-1 expression and the malignancy of GC patients [Supplementary Figure 1A and B]. Moreover, Kaplan-Meier survival analysis underscored that high Fra-1 expression correlated with shortened overall survival (OS) and disease-free survival (DFS) in patients [Figure 1B, Supplementary Figure 1C], underscoring its association with poor prognosis in GC. Furthermore, through a comprehensive examination of three drug-resistant GC datasets in the GEO database, we discerned that Fra-1 expression was prevalent in chemotherapy-resistant GC [Figure 1C], suggesting a potential link between Fra-1 and chemotherapy resistance in GC. This association was further validated through *in vitro* cell experiments. Specifically, we modulated Fra-1 expression levels in GC cells AGS and HGC27 and subjected them to varying concentrations of CDDP. Subsequent assessment of cell proliferation via the cell counting kit-8 (CCK8) kit after 24 h revealed a dose-dependent reduction in proliferative capacity in CDDP-treated GC cells. Notably, compared to the negative control (NC) group, the Fra-1 overexpression group exhibited a slower decline in proliferative capacity and diminished sensitivity to CDDP, suggesting that elevated Fra-1 expression may attenuate GC cell sensitivity to CDDP (*P* < 0.01). Conversely, Fra-1 silencing elicited an opposing trend [Figure 1D-G, Supplementary Figure 1D]. To corroborate these findings, we employed the 5-ethynyl-2’-deoxyuridine (EDU) cell proliferation assay, which similarly demonstrated a significant reduction in the number of proliferating cells following CDDP treatment in AGS and HGC27 GC cells. Importantly, the Fra-1 overexpression group exhibited a higher number of proliferating cells, indicative of resistance to CDDP, compared to the NC group. Conversely, Fra-1 silencing yielded results contrary to Fra-1 overexpression(*P* < 0.05) [Figure 1H-L]. Collectively, our findings underscore the pivotal role of elevated Fra-1 expression in mediating chemoresistance in GC cells.

### Fra-1 induces chemoresistance in GC cells by activating the PPP metabolic pathway

**Figure 2:**
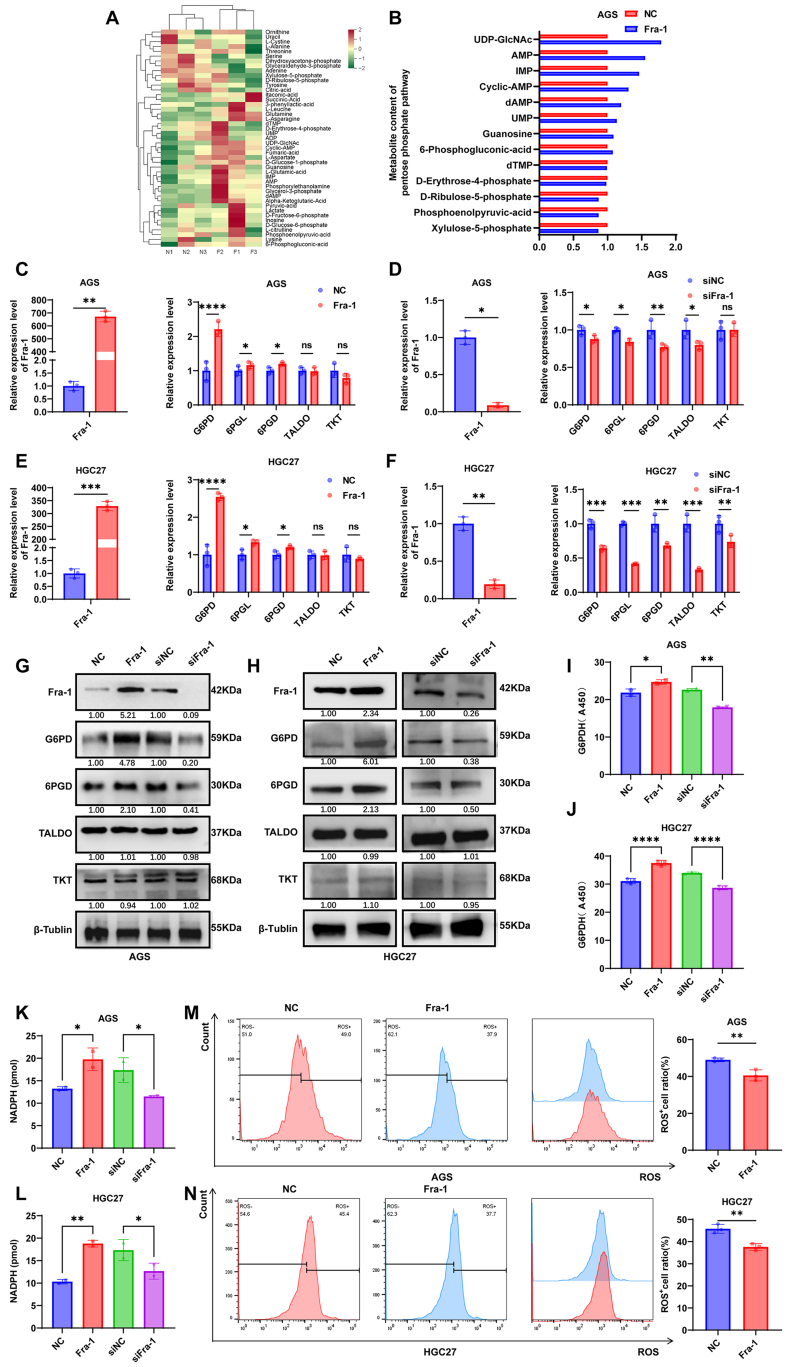
Fra-1 activates the PPP pathway in GC cells. (A) Heatmap showing the relative abundance of non-targeted metabolites in the NC group versus the Fra-1 overexpression group in GC cells (*n* = 6); (B) Expression levels of PPP metabolites; (C-F) RT-qPCR analysis of key molecules in the PPP metabolism pathway after overexpression/knockdown of Fra-1 in GC cells AGS and HGC27; (G and H) Protein levels of key molecules in the metabolic pathway of the PPP after overexpression/knockdown of Fra-1 in GC cells AGS and HGC27 detected by western blot assay; (I and J) G6PD enzyme activity measured after overexpression/knockdown of Fra-1 in GC cells AGS and HGC27 using the G6PD enzyme activity kit; (K and L) NADPH content measured after overexpression/knockdown of Fra-1 in GC cells AGS and HGC27 using the NADPH kit; (M and N) ROS content measured after overexpression/knockdown of Fra-1 in GC cells AGS and HGC27 using the ROS kit. All experiments were performed with three technical replicates. ^*^P < 0.05; ^**^P < 0.01; ^***^P < 0.001; ^****^P < 0.0001. Fra-1: Fos-related antigen-1; PPP: pentose phosphate pathway; GC: gastric cancer; G6PD: glucose-6-phosphate dehydrogenase; NADPH: nicotinamide adenine dinucleotide phosphate; ROS: reactive oxygen species.

To elucidate the intricate mechanism underlying Fra-1-mediated chemoresistance in GC cells, we initiated Fra-1 overexpression in AGS GC cells, followed by energy metabolomics analyses. These analyses unveiled a notable impact of Fra-1 on the energy metabolic pathway within GC cells. Further exploration through differential metabolite analyses revealed a significant upregulation of metabolites associated with the PPP in Fra-1-overexpressing GC cells [Figure 2A and B, Supplementary Figure 1E].

To corroborate the aforementioned metabolomics findings. Firstly, we employed RT-qPCR to analyze the mRNA levels of key molecules involved in PPP metabolic pathway. The results unveiled a significant upregulation of mRNA levels of the oxidative branch key enzymes G6PD, 6-phosphogluconolactonase (6PGL), and 6-phosphogluconate dehydrogenase (6PGD) in Fra-1-overexpressing GC cells, while no statistically significant differences were observed in the mRNA levels of the non-oxidative branch key enzymes transaldolase (TALDO) and transketolase (TKT). Conversely, the knockdown of Fra-1 led to a decrease in the mRNA levels of the aforementioned oxidative branch key enzymes in both AGS and HGC27 cells, with notable reductions observed in 6PGL and 6PGD mRNA levels [Figure 2C-F]. Moreover, western blot analysis demonstrated a significant increase in the protein levels of G6PD and 6PGD upon Fra-1 overexpression, whereas their protein levels were notably reduced upon Fra-1 knockdown [Figure 2G and H, Supplementary Figure 1F and G]. We further investigated whether Fra-1 influences G6PD enzymatic activity. We observed a significant elevation in G6PD enzyme activity upon Fra-1 overexpression, while Fra-1 knockdown led to a decrease in G6PD enzyme activity, displaying a positive correlation with G6PD protein levels [Figure 2I and J]. Our findings revealed that Fra-1 overexpression promoted the production of NADPH in the PPP metabolic pathway and decreased intracellular ROS levels, whereas silencing of Fra-1 resulted in decreased NADPH production and elevated ROS levels in GC cells (*P <* 0.05) [Figure 2K-N, Supplementary Figure 1H and I]. These results collectively indicate that Fra-1 activation triggers the PPP metabolic pathway in GC cells.

**Figure 3:**
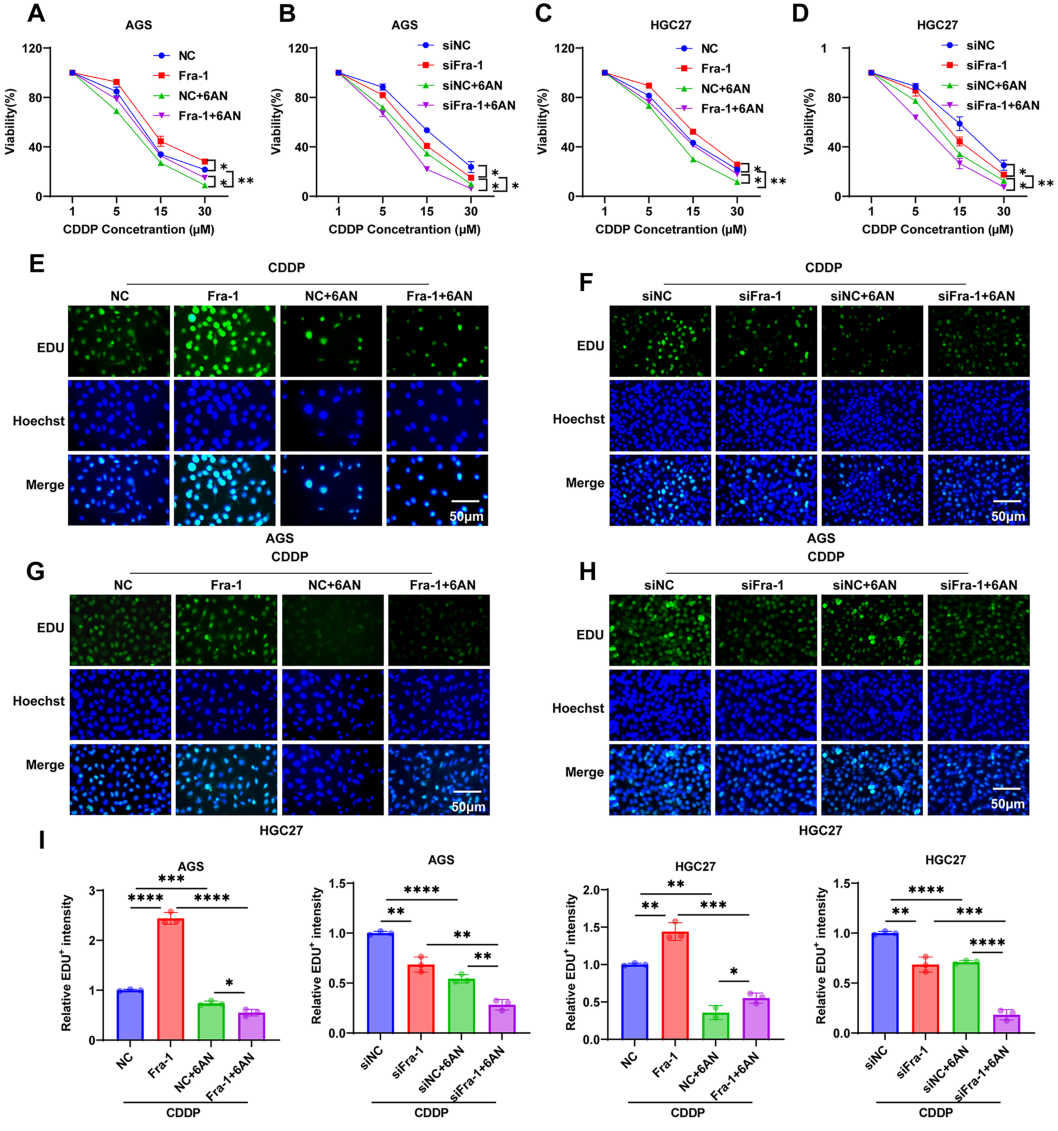
Fra-1 induces chemoresistance in GC cells by activating the PPP pathway. (A-D) Cell proliferation ability of GC cells after overexpression/silencing of Fra-1, treatment with PPP metabolism pathway inhibitor 6AN (4 μM), and treatment with different concentrations of CDDP detected using the CCK8 kit; (E-H) Cell proliferation ability of GC cells after overexpression/silencing of Fra-1 and treatment with 6AN (4 μM), and treatment with CDDP (10 μM) detected using the EDU cell proliferation assay kit; (I) Statistical graph of semi-quantitative results of EDU fluorescence values of GC cells. All experiments were performed with three technical replicates. ^*^P < 0.05; ^**^P < 0.01; ^***^P < 0.001; ^****^P < 0.0001. Fra-1: Fos-related antigen-1; GC: gastric cancer; PPP: pentose phosphate pathway; CDDP: cisplatin; CCK8: cell counting kit-8; EDU: 5-ethynyl-2’-deoxyuridine.

Meanwhile, we found that compared to the NC group, the Fra-1 overexpression group exhibited a slower decrease in cell proliferation and lower sensitivity to CDDP. Importantly, the proliferation of GC cells significantly decreased upon overexpression of Fra-1 combined with 6AN treatment compared to the Fra-1 overexpression group alone. Moreover, the proliferation of GC cells was further reduced following Fra-1 silencing combined with 6AN treatment compared to silencing Fra-1 alone(*P* < 0.01) [Figure 3A-D, Supplementary Figure 1J]. Furthermore, we corroborated these findings using the EDU cell proliferation assay. Consistently, the proportion of proliferating cells was higher in the Fra-1 overexpression group, indicating tolerance to CDDP compared to the NC group. Remarkably, the proportion of proliferating cells significantly decreased in the Fra-1 overexpression group concurrently treated with 6AN compared to Fra-1 overexpression alone. Furthermore, the proportion of proliferating cells was further reduced in the Fra-1 silencing group concurrently treated with 6AN (4 μM) compared to Fra-1 silencing alone(*P* < 0.05) [Figure 3E-I]. In summary, these findings strongly suggest that Fra-1 induces chemoresistance in GC cells by activating the PPP metabolic pathway.

### Fra-1 binds to the promoter of G6PD to regulate its transcription and thus activate the PPP metabolic pathway

**Figure 4:**
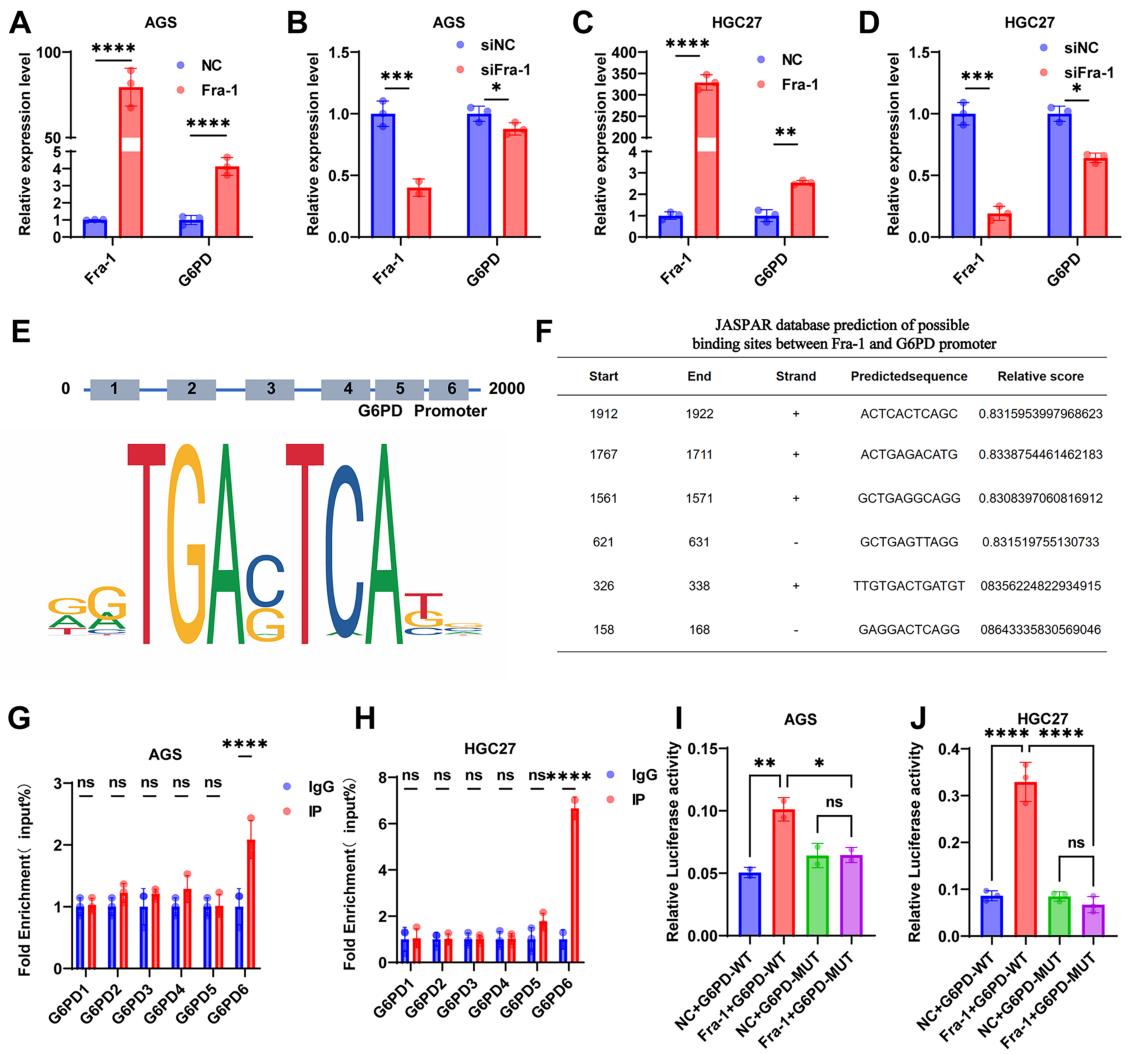
Fra-1 binds to the G6PD promoter and transcriptionally regulates its expression. (A-D) Effects of overexpression/silencing of Fra-1 on G6PD mRNA levels in GC cells AGS and HGC27 detected by RT-qPCR assay; (E) Schematic diagram of G6PD promoter binding motif with Fra-1 DNA; (F) Prediction of possible binding sites of Fra-1 with the G6PD promoter using the JASPAR database; (G and H) Validation of the binding site of Fra-1 to G6PD using ChIP-qPCR experiments; (I and J) Detection of the binding of Fra-1 to the 6th binding site of G6PD using wild-type and mutant dual luciferase reporter gene vectors for the 6th binding site of G6PD in GC cells AGS and HGC27. All experiments were performed with three technical replicates. ns, no significant difference. ^*^P < 0.05; ^**^P < 0.01; ^***^P < 0.001; ^****^P < 0.0001. Fra-1: Fos-related antigen-1; G6PD: glucose-6-phosphate dehydrogenase; GC: gastric cancer.

Through RT-qPCR assays, we observed that the mRNA level of G6PD was upregulated in GC cells upon overexpression of Fra-1 and downregulated upon silencing of Fra-1 [Figure 4A-D], indicating that Fra-1 positively regulates G6PD mRNA expression. Consequently, we hypothesized that Fra-1 may activate PPP metabolism by directly modulating G6PD expression. To explore this hypothesis, we initially predicted potential binding sites of Fra-1 within the G6PD promoter using the JASPAR database and identified six putative binding sites [Figure 4E and F]. To validate the binding of Fra-1 to the G6PD promoter region, we synthesized primers targeting the identified binding sites and conducted ChIP-qPCR assays. Our findings revealed that Fra-1 binds to the 6th site of the G6PD promoter in GC cells AGS and HGC27(*P <* 0.0001) [Figure 4G and H]. To further corroborate this result, we constructed dual-luciferase reporter gene vectors containing the wild-type and mutant sequences of the 6th binding site of the G6PD promoter. Subsequently, these vectors were transfected into GC cells AGS and HGC27. The luciferase activity assay demonstrated that the luciferase activity was significantly elevated in the Fra-1 overexpression group compared to the NC group in the wild-type dual-luciferase reporter gene vector containing the 6th binding site of the G6PD promoter. However, this enhanced luciferase activity was not observed in the Fra-1 overexpression group when the 6th site of the G6PD promoter was mutated, indicating that Fra-1 binding to the G6PD promoter is dependent on the integrity of the 6th binding site(*P <* 0.01) [Figure 4I and J]. These results unequivocally confirm that Fra-1 upregulates G6PD transcription by directly binding to the 6th site of the G6PD promoter region.

### Fra-1 inhibits G6PD ubiquitination, stabilizes its protein level and activates the PPP metabolic pathway

**Figure 5:**
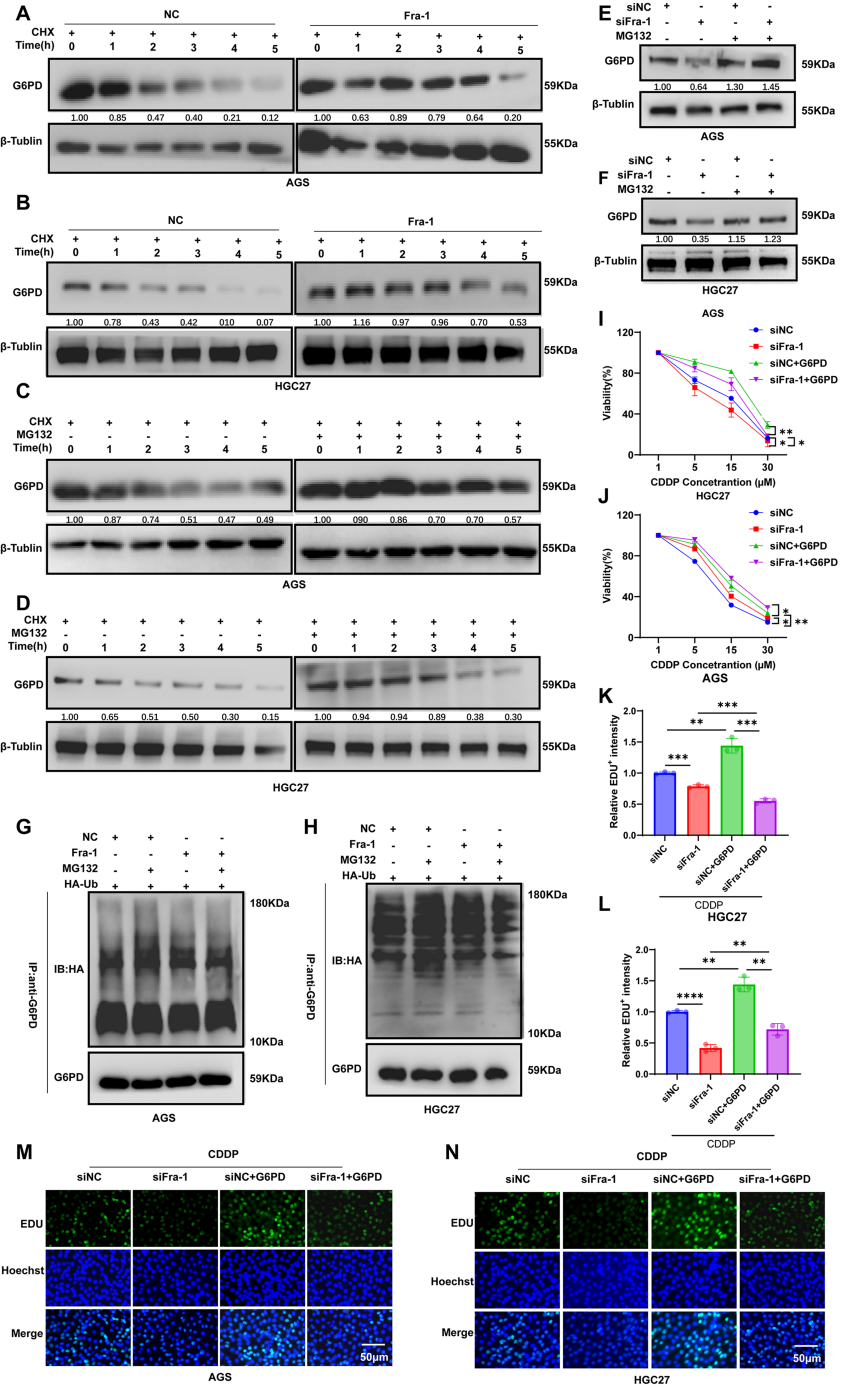
Fra-1 stabilizes G6PD protein expression levels and activates the PPP metabolic pathway by inhibiting the G6PD ubiquitin-proteasome pathway. (A and B) Overexpression of Fra-1 in GC cells AGS and HGC27, followed by treatment with the protein synthesis inhibitor CHX for various durations, and detection of G6PD protein expression levels using western blot assay; (C and D) Treatment of GC cells AGS and HGC27 with CHX for various durations, with the addition of the proteasome pathway inhibitor MG132 in the experimental group, followed by detection of G6PD protein expression levels using western blot assay; (E and F) Silencing of Fra-1 in GC cells AGS and HGC27, followed by treatment with MG132, and detection of G6PD protein expression levels using western blot assay; (G and H) Co-transfection of Fra-1 plasmid and ubiquitin plasmid with HA tag (HA-Ub) into GC cells AGS and HGC27, followed by treatment with MG132, and detection of G6PD ubiquitination using immunoprecipitation assay; (I and J) Detection of cell proliferation ability in GC cells AGS and HGC27 after silencing Fra-1, silencing Fra-1 while overexpressing G6PD, and treatment with CDDP for 24 h using the CCK8 Cell Proliferation Detection Kit; (K-N) Detection of cell proliferation ability in GC cells AGS and HGC27 after silencing Fra-1, silencing Fra-1 while overexpressing G6PD, and treatment with CDDP (10 μM) for 24 h using the EDU cell proliferation detection kit. All experiments were performed with three technical replicates. ^*^P < 0.05; ^**^P < 0.01; ^***^P < 0.001. Fra-1: Fos-related antigen-1; G6PD: glucose-6-phosphate dehydrogenase; PPP: pentose phosphate pathway; GC: gastric cancer; CHX: cycloheximide; CDDP: cisplatin; CCK8: cell counting kit-8; cell counting kit-8; EDU: 5-ethynyl-2’-deoxyuridine.

The impact of Fra-1 on G6PD extends beyond its transcriptional regulation; it also influences G6PD protein levels and stability through mechanisms independent of its binding to the G6PD promoter. To investigate this further, we overexpressed Fra-1 in GC cells AGS and HGC27 and treated them with the protein synthesis inhibitor cycloheximide (CHX). Subsequently, western blot assays revealed that in the absence of CHX treatment (NC group), the expression of G6PD protein began to decline after 1 h, progressively decreasing with prolonged CHX exposure, indicating intracellular degradation of G6PD protein. Conversely, following Fra-1 overexpression, the rate of G6PD degradation was notably attenuated. Specifically, degradation commenced after 3 h of CHX treatment, and the stability of G6PD protein was substantially higher compared to the NC group. These findings suggest that Fra-1 may enhance the stability of G6PD protein by inhibiting its degradation [Figure 5A and B, Supplementary Figure 1K and L].

To further investigate whether G6PD protein degradation relies on the ubiquitin-proteasome pathway, we treated GC cells AGS and HGC27 with CHX while concurrently adding the MG132 for 4 h. Our results demonstrated that in the presence of MG132, the stability of G6PD proteins was notably enhanced compared to the NC group, with a significant reduction in the rate of protein degradation [Figure 5C and D, Supplementary Figure 1M and N]. These findings suggest that G6PD degradation is dependent on the proteasome pathway. Additionally, we silenced Fra-1 in AGS and HGC27 cells and treated them with MG132. Remarkably, the knockdown of Fra-1 led to a reduction in G6PD protein expression, which was restored upon the addition of MG132 [Figure 5E and F, Supplementary Figure 1O and P], providing further evidence that Fra-1 stabilizes G6PD protein expression by modulating the proteasome pathway. These results solidify the notion that Fra-1 regulates G6PD protein stability through the proteasome pathway. Protein degradation via the proteasome pathway often involves ubiquitin protein binding. To ascertain whether G6PD interacts with ubiquitin proteins, we transfected Fra-1 plasmid and HA-Ub into AGS and HGC27 cells, followed by MG132 treatment after 48 h. Subsequently, immunoprecipitation (IP) experiments revealed that G6PD bound to ubiquitin proteins in the NC group following concurrent MG132 treatment. Notably, the proportion of G6PD binding to ubiquitin proteins was significantly diminished after Fra-1 overexpression coupled with MG132 treatment [Figure 5G and H]. These results strongly suggest that G6PD undergoes degradation via the proteasome-dependent pathway through ubiquitin binding. In summary, our findings indicate that Fra-1 inhibits the ubiquitination of G6PD, thereby stabilizing its protein level and highlighting the regulatory role of Fra-1 in modulating G6PD protein degradation through the ubiquitin-proteasome pathway.

To further elucidate the role of G6PD in mediating the effects of Fra-1 on chemoresistance in GC cells, we silenced Fra-1 and simultaneously overexpressed G6PD in AGS and HGC27 cells. Subsequently, the cells were treated with varying concentrations of CDDP, and their proliferative ability was assessed using a CCK8 kit. Our results demonstrated that the proliferation ability of cells in the siFra-1 group decreased more rapidly, indicating higher sensitivity to CDDP compared to the siNC group. Moreover, compared to the siNC group, the siFra-1 group exhibited a faster decline in proliferative capacity and increased sensitivity to CDDP. Conversely, after silencing Fra-1 and overexpressing G6PD, the proliferation ability of GC cells significantly increased compared to the siFra-1 group [Figure 5I and J]. These findings suggest that Fra-1 may activate the PPP metabolic pathway by stabilizing the protein level of G6PD, consequently reducing the cell proliferation ability and enhancing resistance to CDDP in GC cells. Furthermore, we validated these results using the EDU cell proliferation assay. In AGS and HGC27 cells, the proportion of proliferating cells in the Fra-1 silencing group exhibited lower sensitivity to CDDP compared to the siNC group. Notably, the proportion of proliferating cells in the group where Fra-1 was silenced while G6PD was overexpressed was significantly higher compared to the siFra-1 group (*P* < 0.01) [Figure 5K-N]. These findings further confirm that Fra-1 diminishes the sensitivity of GC cells to CDDP by activating the PPP metabolic pathway through the stabilization of G6PD protein levels.

### Fra-1 inhibits ferroptosis in GC cells and induces chemotherapy resistance by activating the PPP metabolic pathway

**Figure 6:**
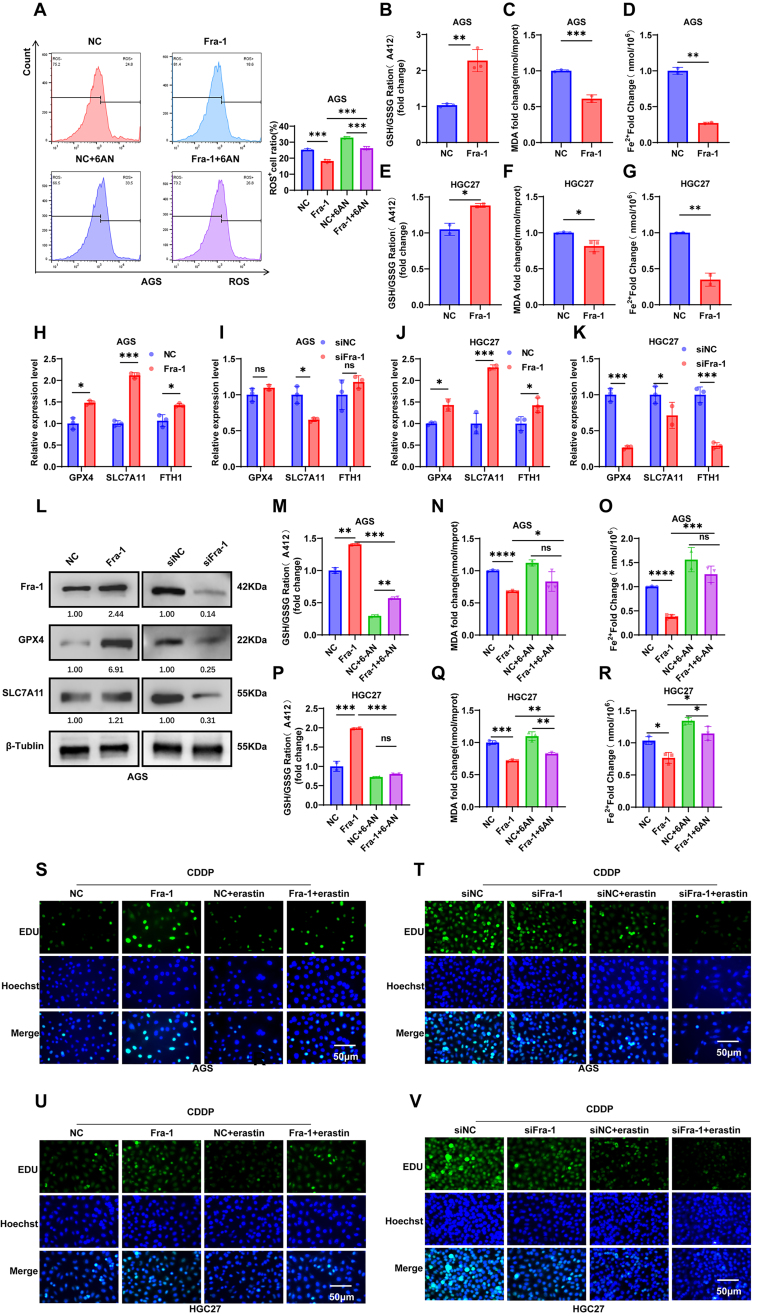
Fra-1 inhibits ferroptosis and induces chemoresistance in GC cells by activating the PPP metabolic pathway. (A) Detection of ROS levels in GC cells AGS after overexpression of Fra-1, and overexpression of Fra-1 along with treatment with 6AN (4 μM), using a ROS detection kit; (B-G) Detection of the effect of Fra-1 on GSH, MDA, Fe^2+^ levels in GC cells AGS and HGC27 using GSH, MDA, Fe^2+^ ELISA assay kit after overexpression/silencing of Fra-1; (H-K) RT-qPCR assay to detect mRNA expression levels of ferroptosis negatively related factors GPX4, SLCA7A11, and FTH1 in GC cells AGS and HGC27 after overexpression/silencing of Fra-1; (L) Detection of protein expression levels of ferroptosis negatively related factors GPX4 and SLCA7A11 in GC cells AGS and HGC27 after overexpression/silencing of Fra-1 using western blot assay; (M-R) Detection of the effect of Fra-1 on GSH, MDA, Fe^2+^ content in GC cells AGS and HGC27 after overexpression of Fra-1, and overexpression of Fra-1 along with treatment with 6AN (4 μM), using GSH, MDA, Fe^2+^ ELISA assay kit; (S-V) Detection of cell proliferation ability in GC cells AGS and HGC27 after overexpression/silencing of Fra-1, overexpression/silencing of Fra-1 along with the addition of ferroptosis inducer erastin (8 μM), and treatment with CDDP (10 μM) for 24 h using the EDU Cell Proliferation Detection Kit. All experiments were performed with three technical replicates. ns, no significant difference; ^*^P < 0.05; ^**^P < 0.01; ^***^P < 0.001. Fra-1: Fos-related antigen-1; GC: gastric cancer; PPP: pentose phosphate pathway; ROS: reactive oxygen species; GSH: glutathione; MDA: malondialdehyde; CDDP: cisplatin; EDU: 5-ethynyl-2’-deoxyuridine.

After elucidating the specific mechanism by which Fra-1 activates the PPP pathway, we aimed to delve deeper into how Fra-1 activation of the PPP pathway induces chemotherapy resistance in GC cells. We, using a ROS detection kit combined with flow cytometry analysis, observed that the ROS levels in GC cells overexpressing Fra-1 decreased, while ROS levels recovered after treatment with 6AN (*P* < 0.001) [Figure 6A, Supplementary Figure 1Q and R]. This indicates that Fra-1 reduces intracellular ROS levels by activating the PPP metabolic pathway, thereby shielding cells from oxidative stress damage. Hence, we hypothesized whether Fra-1-induced activation of the PPP pathway inhibits ferroptosis, consequently inducing chemotherapy resistance in GC cells. To test this hypothesis, we first analyzed the correlation between Fra-1 expression and ferroptosis-related molecules GPX4, SLC7A11, and FTH1 in GC cells through the GEPIA2 website using STAD correlation data in TCGA, and the results showed that Fra-1 was significantly and positively correlated with GPX4, SLC7A11, and FTH1. The results suggested that Fra-1 might be closely related to ferroptosis in GC cells [Supplementary Figure 1S-U]. Next, we overexpressed Fra-1 in GC cells and examined its effect on ferroptosis. The results revealed that Fra-1 overexpression led to an increase in glutathione (GSH) content and a decrease in the levels of malondialdehyde (MDA) and ferrous ions (Fe^2+^). Conversely, knocking down Fra-1 reduced GSH content and increased MDA and Fe^2+^ levels [Figure 6B-G, Supplementary Figure 2A-F]. Overall, Fra-1 overexpression inhibited ferroptosis in GC cells. Further, RT-qPCR and Western blot experiments demonstrated that Fra-1 overexpression upregulated the mRNA and protein expression levels of key molecules such as GPX4, SLCA7A11, and FTH1. Conversely, silencing Fra-1 led to the downregulation of these key molecules [Figure 6H-L, Supplementary Figure 2G-I], further corroborating Fra-1’s role in inhibiting ferroptosis in GC cells. Meanwhile, we used electron microscopy to further observe the effect of Fra-1 on mitochondria in GC cells. The results showed that silencing of Fra-1 resulted in the mitochondrial membrane becoming dense, mitochondrial volume becoming smaller and mitochondrial cristae decreasing, which was consistent with the changes in mitochondrial morphology in cells after the occurrence of ferroptosis. The results further confirmed that Fra-1 overexpression inhibited ferroptosis in GC cells [Supplementary Figure 2J and K]. To further verify that Fra-1 affects the production of GSH, MDA, and Fe^2+^ in GC cells by inhibiting their ferroptosis, we silenced Fra-1 in GC cells AGS and HGC27, both with and without treatment with Ferrostatin-1 (Fer, 1 μM), a ferroptosis inhibitor. The results showed that silencing Fra-1 in these cells led to a decrease in GSH content and an increase in MDA and Fe^2+^ content, compared to the NC group. However, when silencing Fra-1 along with Fer treatment resulted in a significant increase in GSH content and a decrease in MDA and Fe^2+^ content compared to silencing Fra-1 alone. These findings further indicated that Fra-1 in GC cells led to an increase in GSH content and a decrease in MDA and Fe^2+^ content by inhibiting their ferroptosis [Supplementary Figure 2L-Q]. Additionally, we assessed GSH, MDA, and Fe^2+^ levels in GC cells after overexpressing/silencing Fra-1 and treating them with the PPP metabolic pathway inhibitor 6AN. The results demonstrated that overexpression of Fra-1 increased intracellular GSH content and decreased MDA and Fe^2+^ content. However, co-treatment with 6AN reversed these effects. Similarly, silencing Fra-1 reduced intracellular GSH content and increased MDA and Fe^2+^ content, which was further exacerbated by co-treatment with 6AN [Figure 6M-R, Supplementary Figure 2R-V]. Through these experiments, we confirmed that Fra-1 inhibits ferroptosis in GC cells by activating the PPP metabolic pathway. This sheds light on the mechanism by which Fra-1 induces chemotherapy resistance in GC cells.

To elucidate whether Fra-1 mediates chemotherapy resistance in GC cells by suppressing ferroptosis, we modulated Fra-1 expression levels and administered erastin, a ferroptosis inducer, in conjunction with CDDP treatment. Employing EDU cell proliferation assays alongside flow cytometry analysis, we observed a notable increase in the proliferation rate of GC cells overexpressing Fra-1 compared to the NC group upon exposure to CDDP, indicative of reduced sensitivity to CDDP. Conversely, cells overexpressing Fra-1 and treated with erastin exhibited a substantial decrease in proliferation and heightened sensitivity to CDDP compared to the Fra-1 overexpression group alone. Furthermore, silencing Fra-1 led to a significant reduction in cell proliferation relative to the NC group, accompanied by heightened sensitivity to CDDP. Interestingly, simultaneous treatment with erastin and Fra-1 silencing further diminished cell proliferation and enhanced sensitivity to CDDP compared to Fra-1 silencing alone(*P* < 0.05) [Figure 6S-V, Supplementary Figure 2W and X]. These findings collectively suggest that Fra-1 promotes chemotherapy resistance in GC cells by suppressing ferroptosis.

### *In vivo* experiments further confirm that Fra-1 affects chemoresistance and tumor progression in GC cells through activation of the PPP pathway

**Figure 7:**
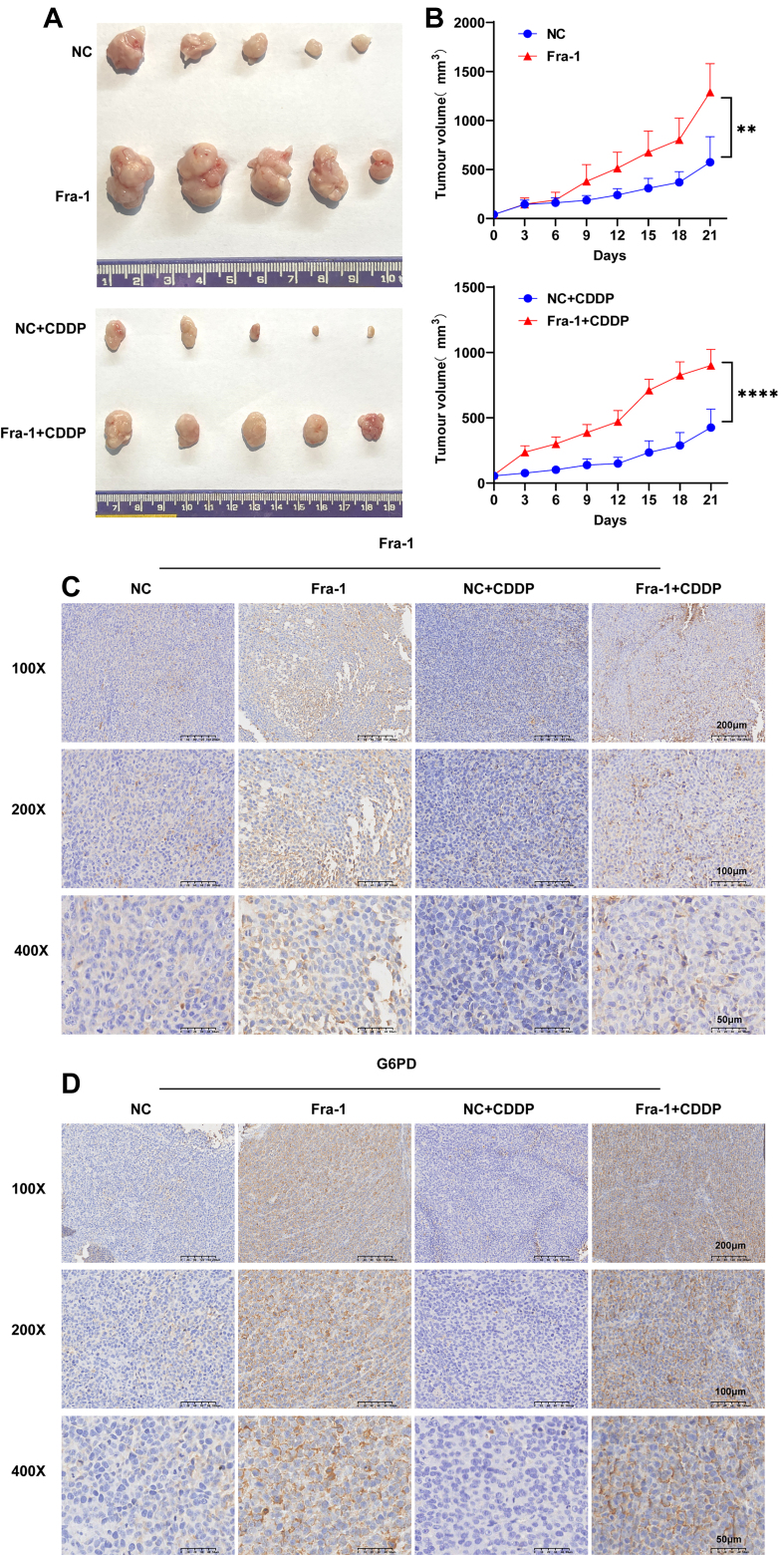
*In vivo* experiments confirmed that Fra-1 promotes GC proliferation. (A and B) Twenty 4-week-old female B/C nude mice were randomly divided into four groups: null group, Fra-1 overexpression group, null + CDDP group, and Fra-1 overexpression + CDDP group, each containing five nude mice. GC cells HGC27 overexpressing Fra-1 were inoculated into the right axilla of each mouse. Tumor size was measured every 3 days using the formula: length × width^2^ × 0.5. When the tumor volume reached 10 mm^3^, CDDP solution (4 mg/kg) was injected intraperitoneally once a week. Three weeks after injection, the nude mice were euthanized, and subcutaneous tissues were collected and photographed; (C and D) Paraffin-embedded GC tumor tissues from nude mice were sectioned and subjected to immunohistochemistry experiments to detect the expression of Fra-1 and G6PD. 100×, Scale bar = 200 μm; 200×, Scale bar = 100 μm; 400×, Scale bar = 50 μm. All experiments were performed with three technical replicates. Statistical analysis was performed to determine significance levels (^**^P < 0.01; ^****^P < 0.0001). Fra-1: Fos-related antigen-1; GC: gastric cancer; CDDP: cisplatin; G6PD: glucose-6-phosphate dehydrogenase.

To further validate the impact of Fra-1 on chemoresistance and tumor progression in GC cells, we conducted *in vivo* experiments. Female B/C nude mice were utilized as study subjects. Subcutaneous injection of 5 × 10^6^ GC HGC27 cells stably overexpressing Fra-1 into the right axilla of each nude mouse initiated tumor formation. Following three weeks of treatment, the mice were euthanized, and tumor tissues were excised, photographed, and recorded. Our findings revealed that the Fra-1 overexpression group exhibited larger tumor volumes and accelerated growth rates compared to the null group. CDDP treatment significantly reduced tumor volume; however, the Fra-1 + CDDP group displayed larger tumor volumes, enhanced growth rates, and decreased sensitivity to CDDP compared to the null + CDDP group (*P* < 0.01) [Figure 7A and B]. These outcomes suggest that Fra-1 overexpression promotes GC proliferation and chemoresistance. To further investigate whether Fra-1 induces chemoresistance in GC cells *in vivo* by modulating the PPP metabolic pathway, we performed immunohistochemistry on paraffin-embedded sections of transplanted tumor tissues from nude mice to assess Fra-1 and G6PD expression. Results revealed elevated Fra-1 expression in tumor tissues from the Fra-1 overexpression group and Fra-1 overexpression + CDDP group compared to the NC group [Figure 7C], accompanied by increased G6PD expression [Figure 7D]. Moreover, RT-qPCR results further showed a positive correlation between the expression levels of Fra-1 and key molecules of the oxidative branch of the PPP pathway (G6PD, 6PGD) in mouse transplanted tumor tissues [Supplementary Figure 2Y]. These findings further support the notion that Fra-1 influences chemoresistance and tumor progression in GC cells by activating the PPP pathway *in vivo*.

## DISCUSSION

GC ranks sixth in incidence rate and third in mortality, making it a significant contributor to cancer-related deaths worldwide^[19]^. Most patients are diagnosed in advanced stages, where chemotherapy becomes a primary treatment modality^[20]^. With the detailed mechanisms of chemotherapy resistance in GC remaining incompletely understood, the prognosis for advanced GC patients remains poor, with low 5-year survival rates^[14,21-23]^. Thus, elucidating the underlying mechanisms of chemotherapy resistance in GC is imperative. Malignant tumor chemotherapy resistance often involves the activation of oncogenes. Fra-1, a member of the Fos family, serves as a crucial oncogene implicated in various biological processes, including cell growth, differentiation, and apoptosis^[24,25]^. In our previous study, we established that Fra-1 significantly influences the biological behavior of GC cells, including apoptosis, thereby implicating its involvement in GC development. Here, we observed high expression of Fra-1 in GC tissue, correlating closely with chemotherapy resistance and poor prognosis. *In vitro* cytology experiments further validated Fra-1’s impact on chemotherapy resistance in GC cells. Our findings underscore the pivotal role of elevated Fra-1 expression in mediating chemoresistance in GC cells. The involvement of Fra-1 in tumor malignancy has been extensively reported in various cancers. For instance, in breast cancer, Fra-1 acts as a regulator of epithelial-mesenchymal transition (EMT) and metastasis, while also enhancing chemical sensitivity^[26-28]^. Similarly, in colorectal cancer, Fra-1 overexpression promotes invasive phenotypes^[29,30]^. Moreover, through mechanisms like the miR-134-SDS22 feedback loop, Fra-1 promotes ERK/JNK signaling, reducing chemical sensitivity in ovarian cancer cells^[31]^. Our study aligns with these findings, highlighting the pivotal role of Fra-1 in chemotherapy resistance in GC cells.

To elucidate the intricate mechanism underlying Fra-1-mediated chemoresistance in GC cells, we initiated Fra-1 overexpression in GC cells, followed by energy metabolomics analyses. The results found that the metabolites related to the PPP were significantly upregulated in fra-1-overexpressing GC cells. PPP, a metabolic pathway that complements glycolysis, is abnormally activated in various malignant tumors. It serves as the primary source of NADPH and plays a crucial role in meeting the synthetic metabolic demands of cancer cells while combating oxidative stress by clearing intracellular ROS through redox reactions^[32-34]^. Several PPP enzymes have emerged as potential targets for cancer treatment^[35-37]^. Research has highlighted the involvement of PPP metabolism in cancer cell chemotherapy resistance processes^[38-40]^. For instance, in breast cancer, Rac1 activation of aldolase A and ERK signaling triggers the non-oxidative PPP, enhancing nucleotide metabolism and shielding breast cancer cells from chemotherapy-induced DNA damage, thereby inducing chemotherapy resistance^[9]^. G6PD, the first key rate-limiting enzyme in PPP metabolism, is often dysregulated, leading to aberrant activation or silencing of PPP metabolic pathways^[33]^. Studies have shown that PIKE-A activation of Akt binds to STAT3, stimulating FYN and inducing STAT3 phosphorylation, thereby enhancing G6PD transcription, activating PPP metabolism, promoting DNA and NADPH synthesis, and inhibiting ROS production, ultimately fostering tumor growth^[41]^. Moreover, HPD, closely linked to G6PD transcription, enhances tyrosine breakdown metabolism, elevating acetyl CoA levels for histone acetylation, and promoting HDAC10 translocation to the cytoplasm through LKB1/AMPK signaling, thereby controlling histone acetylation and boosting G6PD transcription, PPP flux, and tumor progression^[42]^. To further elucidate whether Fra-1 induces chemoresistance in GC cells by activating the PPP metabolic pathway, we conducted a series of experiments in AGS and HGC27 GC cells. Our study demonstrates that high Fra-1 expression activates the PPP metabolic pathway in GC cells, boosting NADPH synthesis and reducing intracellular ROS levels. Additionally, Fra-1 directly binds to the G6PD promoter, transcriptionally activating the PPP metabolic pathway, aligning with previous research. Our findings underscore the role of Fra-1 in inducing chemotherapy resistance in GC cells by activating the PPP metabolic pathway. While our study highlights the significance of metabolic reprogramming in GC cell survival, further investigations are warranted to elucidate the potential involvement of other metabolic pathways in GC chemotherapy resistance.

After elucidating the specific mechanism by which Fra-1 activates the PPP pathway, we aimed to delve deeper into how Fra-1 activation of the PPP pathway induces chemotherapy resistance in GC cells. Our results show that the Fra-1 overexpression inhibited ferroptosis in GC cells. Ferroptosis, a regulated cell death pathway, has been implicated in tumor chemotherapy resistance, with studies revealing a negative correlation between PPP metabolism activation and ferroptosis. Activation of the PPP metabolic pathway leads to the synthesis of reduced NADPH, promoting GSH synthesis and conversion of coenzyme Q10 to coenzyme Q10H2, thereby inhibiting peroxide accumulation. This activation also triggers the GPX4/GSH and FSP1/CoQ10H2 pathways, further suppressing ferroptosis^[43,44]^. The interplay between PPP metabolism and ferroptosis in tumor development is significant, yet research on PPP metabolism’s regulation of ferroptosis remains limited. The results of this study suggest that Fra-1 reduces intracellular ROS levels by activating the PPP metabolic pathway, thereby shielding cells from oxidative stress damage. Prior research has established PPP as an effective pathway for clearing ROS, and ferroptosis is closely linked to intracellular oxidation levels. Hence, we hypothesized whether Fra-1-induced activation of the PPP pathway inhibits ferroptosis, consequently inducing chemotherapy resistance in GC cells. Our study reveals that Fra-1 indeed inhibits ferroptosis in GC cells by activating the PPP metabolic pathway, consequently inducing chemotherapy resistance. This finding was corroborated by *in vivo* experiments in nude mice. However, further investigation is warranted to elucidate the detailed mechanism of ferroptosis-induced chemotherapy resistance in GC cells, including whether it involves classical ferroptosis-related pathways such as GPX4 and SLC7A11. In summary, our findings demonstrate that Fra-1 binds to the G6PD promoter, transcriptionally regulating its expression, and inhibits G6PD ubiquitination degradation by suppressing the ubiquitin-proteasome pathway, thereby stabilizing G6PD protein levels. This activation of the PPP metabolic pathway inhibits ferroptosis in GC cells, ultimately inducing chemotherapy resistance. Moreover, we found a short OS in GC patients with high Fra-1 expression by Kaplan-Meier survival analysis, indicating that the expression level of Fra-1 has a close correlation with the prognosis of GC patients. Meanwhile, in order to further verify the correlation between high Fra-1 expression and GC progression through *in vivo* experiments, we performed subcutaneous tumor formation experiments in nude mice. The results showed that Fra-1-overexpressing GC cells had significantly increased volume and fast growth rate. This result further confirmed that Fra-1 overexpression is able to promote GC proliferation and may be a new potential prognostic marker in GC patients.

In this study, we used 6AN, an inhibitor of the PPP, to detect its effect on Fra-1-mediated chemoresistance in GC cells *in vitro*. The results confirmed that Fra-1-mediated chemoresistance in GC cells was significantly attenuated when the PPP was blocked with 6AN. However, while the *in vitro* experiments yielded promising results, they could not fully replicate the complex interactions in the tumor microenvironment *in vivo*. Therefore, further *in vivo* animal model validation and clinical trial evaluation are still needed to determine whether 6AN can be an effective target for chemoresistance in GC cells with high Fra-1 expression.

In summary, our study confirms that Fra-1 regulates its expression by binding to the G6PD promoter and inhibiting the ubiquitin-proteasome pathway, thus stabilizing the G6PD protein level. This action activates the PPP metabolic pathway, leading to increased synthesis of reduced equivalent NADPH, decreased intracellular ROS levels, and inhibition of ferroptosis in GC cells, ultimately inducing chemotherapy resistance. These findings position Fra-1 as a novel regulatory factor in chemotherapy resistance for GC, highlighting its role in mediating G6PD expression to promote tumor chemoresistance. Consequently, Fra-1 emerges as a promising potential target for the clinical treatment of GC.

## DECLARATIONS

### Availability of data and materials

The rata data supporting the findings of this study are available within this article and its Supplementary Materials. Further data is available from the corresponding authors upon request.

### Copyright

© The Author(s) 2024.
