# Supplementary material for: Fra-1 affects chemotherapy sensitivity by inhibiting ferroptosis in gastric cancer cells
Source: Cancer Drug Resist. 2024 Nov 16;7:44. doi: 10.20517/cdr.2024.101 (PMC11609144; doi:10.20517/cdr.2024.101)
Supplement: Supplementary file 1 [file cdr-7-44-SupplementaryMaterials.pdf]

Supplementary Materials

Fra-1 affects chemotherapy sensitivity by inhibiting ferroptosis in gastric cancer cells

Feng Zeng[1], Jiaying Cao[1], Yan Chen[1], Jingqiong Tang[1,2], Qian He[3], Shan Liao[4], Lin Liang[1], Wentao Li[1], Siyi Liu[1], Gengqiu Luo[5], Yanhong Zhou[1]

[1]Cancer Research Institute, Basic School of Medicine, Central South University, Changsha 410011, Hunan, China.
[2]Department of Geriatrics, The Second Xiangya Hospital of Central South University, Changsha 410011, Hunan, China.
[3]Department of Radiation Oncology, Hunan Cancer Hospital & the Affiliated Cancer Hospital of Xiangya School of Medicine, Central South University, Changsha 410013, Hunan, China.
[4]Department of Pathology, The Third Xiangya Hospital of Central South University, Changsha 410013, Hunan, China.
[5]Department of Pathology, Xiangya Hospital, Basic School of Medicine, Central South University, Changsha 410008, Hunan, China.

Correspondence to: Dr Yanhong Zhou, Cancer Research Institute, Basic School of Medicine, Central South University, 88 Xiangya Road, Changsha 410011, Hunan, China. E-mail: zhouyanhong@csu.edu.cn; Dr Gengqiu Luo, Department of Pathology, Xiangya Hospital, Basic School of Medicine, Central South University, 88 Xiangya Road, Changsha 410008, Hunan, China. E-mail: luogengqiu@csu.edu.cn

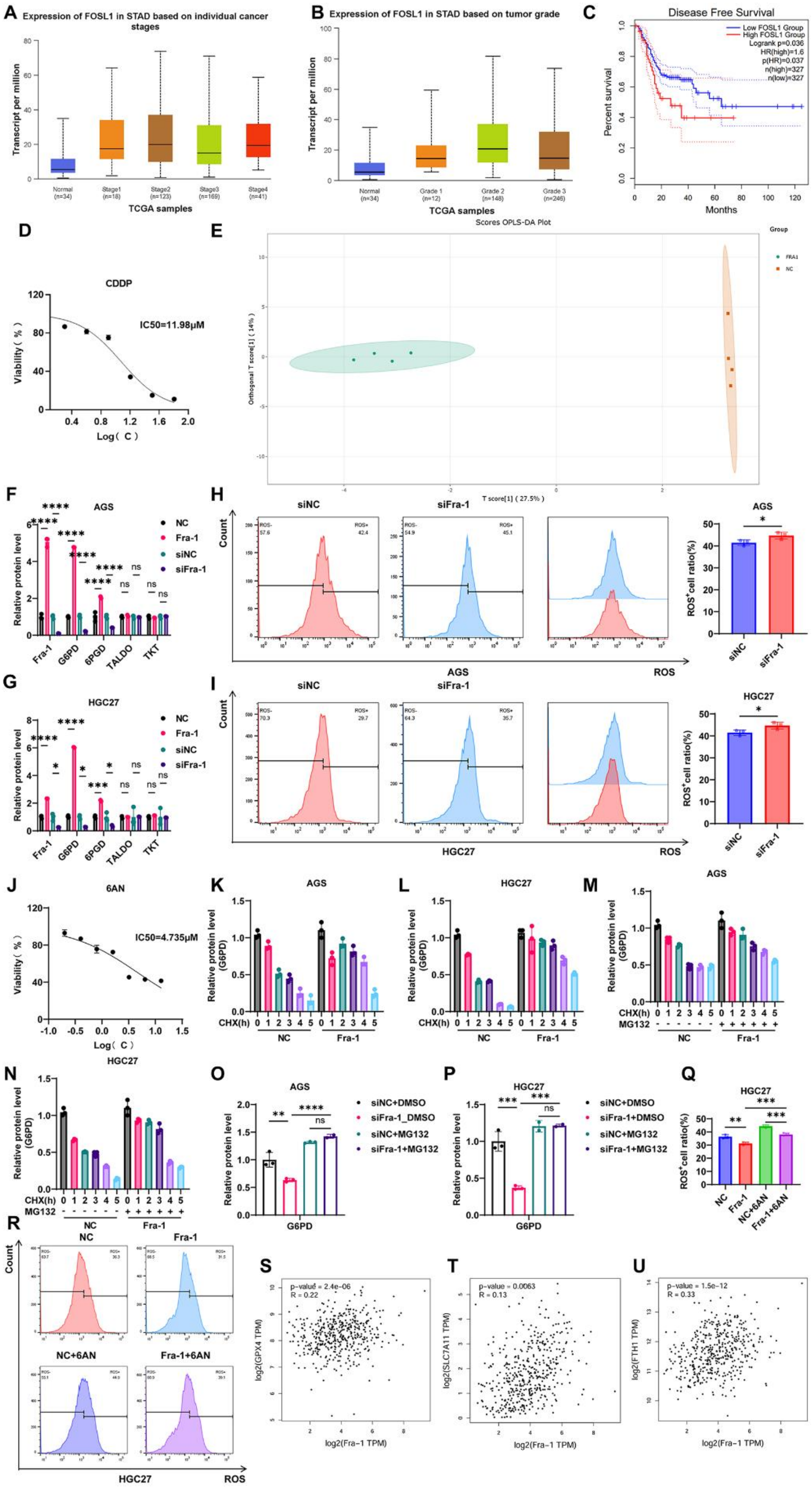
A
Expression of FOSL1 in STAD based on individual cancer stages
Transcript per million
TCGA samples
Normal (n=34)
Stage1 (n=18)
Stage2 (n=123)
Stage3 (n=169)
Stage4 (n=41)
B
Expression of FOSL1 in STAD based on tumor grade
Transcript per million
TCGA samples
Normal (n=34)
Grade 1 (n=12)
Grade 2 (n=148)
Grade 3 (n=246)
C
Disease Free Survival
Low FOSL1 Group
High FOSL1 Group
Logrank p=0.036
HR(high)=1.6
p(HR)=0.037
n(high)=327
n(low)=327
Percent survival
Months
D
CDDP
IC50=11.98μM
Viability（%）
Log（C）
E
Scores OPLS-DA Plot
Group
FRA1
NC
T score[1]（27.5%）
F
AGS
NC
Fra-1
siNC
siFra-1
Relative protein level
Fra-1
G6PD
6PGD
TALDO
TKT
G
HGC27
H
siNC
siFra-1
Count
AGS
ROS
ROS⁺cell ratio(%)
I
HGC27
J
6AN
IC50=4.735μM
K
AGS
Relative protein level (G6PD)
CHX(h)
NC
Fra-1
L
HGC27
M
AGS
MG132
N
HGC27
O
AGS
siNC+DMSO
siFra-1_DMSO
siNC+MG132
siFra-1+MG132
G6PD
P
HGC27
siFra-1+DMSO
Q
HGC27
NC+6AN
Fra-1+6AN
R
NC
Fra-1
NC+6AN
Fra-1+6AN
HGC27
ROS
S
p-value = 2.4e-06
R = 0.22
log2(GPX4 TPM)
log2(Fra-1 TPM)
T
p-value = 0.0063
R = 0.13
log2(SLC7A11 TPM)
U
p-value = 1.5e-12
R = 0.33
log2(FTH1 TPM)

Supplementary Figure 1. Fra-1 affects the PPP metabolic pathway in gastric cancer cells and is closely related to its prognosis. (A and B) Analysis from the DRESIS database evaluates Fra-1 expression across different malignancy degrees of gastric cancer patients in the STAD cohort. (C) The GEPIA2 database examines the relationship between Fra-1 expression and overall survival of gastric cancer patients in the STAD cohort. (D) Determination of IC50 values for CDDP (IC50 = 11.98 μM) in AGS gastric cancer cells. (E) PCA plot analysis showing the relative difference between the NC group and Fra-1 overexpression group in gastric cancer cells. (F and G) Gray scale quantitative statistics of protein bands. (H and I) Assessment of ROS content in AGS and HGC27 gastric cancer cells after silencing Fra-1, performed using a ROS detection kit. (J) Determination of IC50 values for 6AN (IC50 = 4.735 μM) in AGS gastric cancer cells. (K-P) Gray scale quantitative statistics of protein bands. (Q and R) Detection of ROS levels in gastric cancer cell HGC27 after overexpression of Fra-1, and overexpression of Fra-1 along with treatment with 6AN (4 μM), using a ROS detection kit. (S-U) Using STAD-related data from TCGA, analyze the correlation between Fra-1 expression and ferroptosis-related molecules GPX4, SLC7A11, and FTH1 in gastric cancer cells through the GEPIA2 website. All experiments were conducted with three technical replicates. Statistical significance is denoted by $^{*}P < 0.05$; $^{**}P < 0.01$; $^{***}P < 0.001$; $^{****}P < 0.0001$.

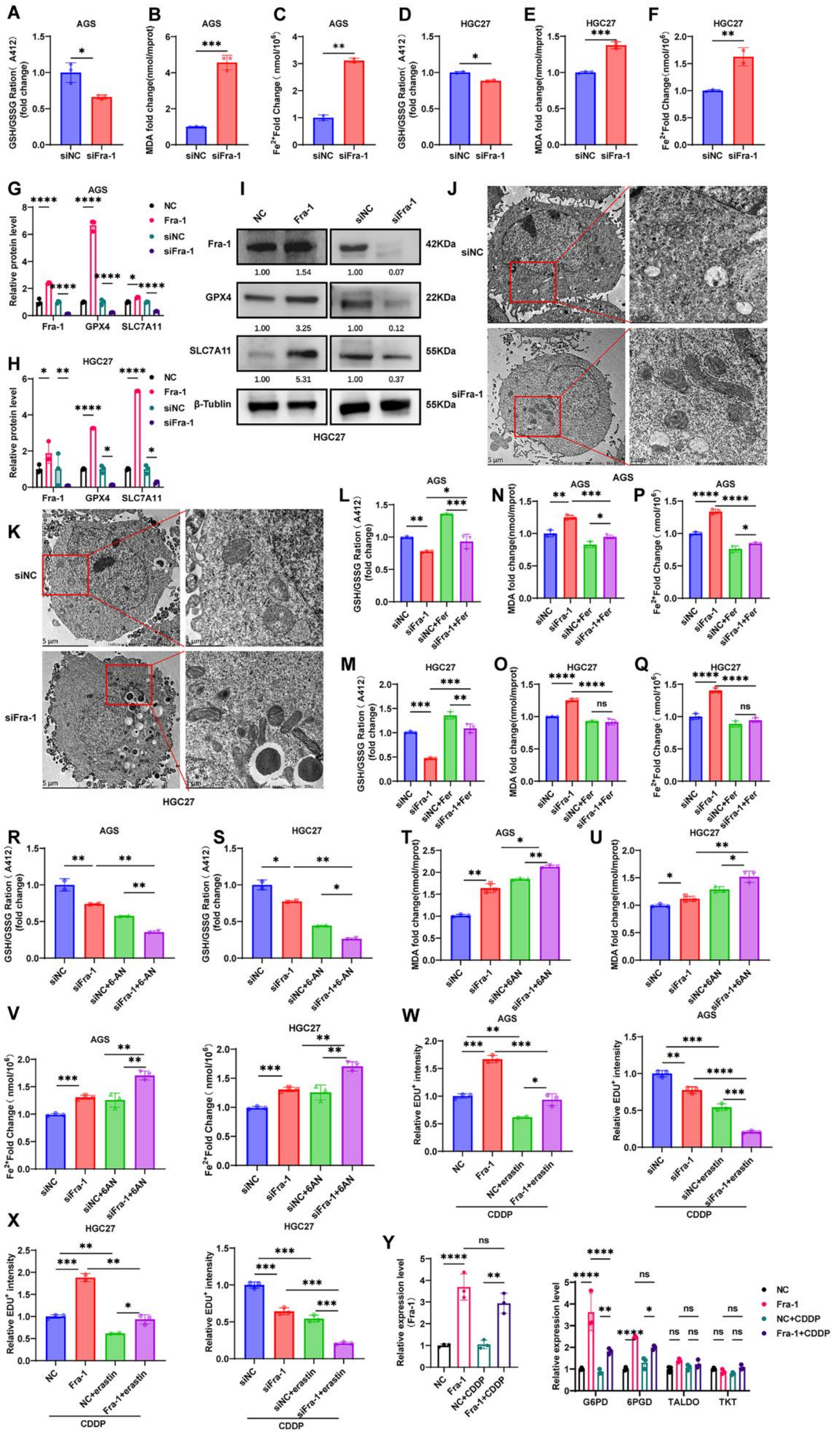

A
AGS
GSH/GSSG Ration(A412)
(fold change)
siNC siFra-1
B
AGS
MDA fold change(nmol/mprot)
siNC siFra-1
C
AGS
Fe2+Fold Change (nmol/10^6)
siNC siFra-1
D
HGC27
GSH/GSSG Ration(A412)
(fold change)
siNC siFra-1
E
HGC27
MDA fold change(nmol/mprot)
siNC siFra-1
F
HGC27
Fe2+Fold Change(nmol/10^6)
siNC siFra-1
G
AGS
Relative protein level
Fra-1 GPX4 SLC7A11
NC
Fra-1
siNC
siFra-1
H
HGC27
Relative protein level
Fra-1 GPX4 SLC7A11
NC
Fra-1
siNC
siFra-1
I
NC Fra-1 siNC siFra-1
Fra-1
42KDa
1.00 1.54 1.00 0.07
GPX4
22KDa
1.00 3.25 1.00 0.12
SLC7A11
55KDa
1.00 5.31 1.00 0.37
β-Tublin
55KDa
HGC27
J
siNC
siFra-1
5 μm
AGS
K
siNC
siFra-1
5 μm
HGC27
L
AGS
GSH/GSSG Ration (A412)
(fold change)
siNC siFra-1 siNC+Fer siFra-1+Fer
M
HGC27
GSH/GSSG Ration (A412)
(fold change)
siNC siFra-1 siNC+Fer siFra-1+Fer
N
AGS
MDA fold change(nmol/mprot)
siNC siFra-1 siNC+Fer siFra-1+Fer
O
HGC27
MDA fold change(nmol/mprot)
siNC siFra-1 siNC+Fer siFra-1+Fer
P
AGS
Fe2+Fold Change (nmol/10^6)
siNC siFra-1 siNC+Fer siFra-1+Fer
Q
HGC27
Fe2+Fold Change (nmol/10^6)
siNC siFra-1 siNC+Fer siFra-1+Fer
R
AGS
GSH/GSSG Ration (A412)
(fold change)
siNC siFra-1 siNC+6-AN siFra-1+6-AN
S
HGC27
GSH/GSSG Ration (A412)
(fold change)
siNC siFra-1 siNC+6-AN siFra-1+6-AN
T
AGS
MDA fold change(nmol/mprot)
siNC siFra-1 siNC+6AN siFra-1+6AN
U
HGC27
MDA fold change(nmol/mprot)
siNC siFra-1 siNC+6AN siFra-1+6AN
V
AGS
Fe2+Fold Change (nmol/10^6)
siNC siFra-1 siNC+6AN siFra-1+6AN
HGC27
Fe2+Fold Change (nmol/10^6)
siNC siFra-1 siNC+6AN siFra-1+6AN
W
AGS
Relative EDU+ intensity
NC Fra-1 NC+erastin Fra-1+erastin
CDDP
AGS
Relative EDU+ intensity
siNC siFra-1 siNC+erastin siFra-1+erastin
CDDP
X
HGC27
Relative EDU+ intensity
NC Fra-1 NC+erastin Fra-1+erastin
CDDP
HGC27
Relative EDU+ intensity
siNC siFra-1 siNC+erastin siFra-1+erastin
CDDP
Y
Relative expression level
(Fra-1)
NC Fra-1 NC+CDDP Fra-1+CDDP
Relative expression level
G6PD 6PGD TALDO TKT
NC
Fra-1
NC+CDDP
Fra-1+CDDP

Supplementary Figure 2. Fra-1 activates the PPP metabolic pathway to inhibit ferroptosis and induce chemoresistance in gastric cancer cells. (A-F) Detection of GSH, MDA, and Fe^{2+} levels in gastric cancer cells following overexpression or silencing of Fra-1, using GSH, MDA, and Fe^{2+} ELISA detection kits. (G and H) Gray scale quantitative statistics of protein bands. (I) Western blot assay examines the protein expression levels of ferroptosis negatively correlated factors GPX4 and SLCA7A11 in HGC27 gastric cancer cells after overexpression or silencing of Fra-1. (J and K) Using electron microscopy to observe the effect of Fra-1 on mitochondria in gastric cancer cells. (L-Q) Evaluation of GSH, MDA, and Fe^{2+} content in gastric cancer cells after silence of Fra-1 and simultaneous treatment with Fer (1 μM), utilizing GSH, MDA, and Fe^{2+} ELISA assay kits. (R-V) Evaluation of GSH, MDA, and Fe^{2+} content in gastric cancer cells after overexpression of Fra-1 and simultaneous treatment with 6AN (4 μM), utilizing GSH, MDA, and Fe^{2+} ELISA assay kits. (W and X) Fluorescence semi-quantitative analysis of cell proliferation ability in AGS and HGC27 gastric cancer cells following overexpression or silencing of Fra-1, combined with the addition of iron-death inducer erastin (8 μM) and treatment with CDDP (10 μM). The analysis was conducted using the EDU Cell Proliferation Detection Kit after 24 h. (Y) The mRNA levels of Fra-1 and key molecules of the PPP metabolic pathway were detected in gastric cancer tissues of four groups of nude mice using RT-qPCR experiments. All experiments were performed with three technical replicates. Statistical significance is indicated by $^{*}P < 0.05$; $^{**}P < 0.01$; $^{***}P < 0.001$; $^{****}P < 0.0001$.
